# Spy&Go purification of SpyTag-proteins using pseudo-SpyCatcher to access an oligomerization toolbox

**DOI:** 10.1038/s41467-019-09678-w

**Published:** 2019-04-15

**Authors:** Irsyad N. A. Khairil Anuar, Anusuya Banerjee, Anthony H. Keeble, Alberto Carella, Georgi I. Nikov, Mark Howarth

**Affiliations:** Department of Biochemistry, University of Oxford, South Parks Road, Oxford, OX1 3QU UK

## Abstract

Peptide tags are a key resource, introducing minimal change while enabling a consistent process to purify diverse proteins. However, peptide tags often provide minimal benefit post-purification. We previously designed SpyTag, forming an irreversible bond with its protein partner SpyCatcher. SpyTag provides an easy route to anchor, bridge or multimerize proteins. Here we establish Spy&Go, enabling protein purification using SpyTag. Through rational engineering we generated SpyDock, which captures SpyTag-fusions and allows efficient elution. Spy&Go enabled sensitive purification of SpyTag-fusions from *Escherichia coli*, giving superior purity than His-tag/nickel-nitrilotriacetic acid. Spy&Go allowed purification of mammalian-expressed, N-terminal, C-terminal or internal SpyTag. As an oligomerization toolbox, we established a panel of SpyCatcher-linked coiled coils, so SpyTag-fusions can be dimerized, trimerized, tetramerized, pentamerized, hexamerized or heptamerized. Assembling oligomers for Death Receptor 5 stimulation, we probed multivalency effects on cancer cell death. Spy&Go, combined with simple oligomerization, should have broad application for exploring multivalency in signaling.

## Abstract (web summary)

Peptide tags are important for protein purification but have minimal benefits afterwards. Here the authors present Spy&Go, which uses rational engineering of the SpyDock protein to capture SpyTag peptides without requiring an irreverisble isopeptide bond.

## Introduction

Affinity chromatography is a central enabling technology for research and for producing therapeutics, vaccines, and diagnostics^1,2^. However, a persistent problem with affinity tags is paradoxically the tags themselves, since the tags often serve no purpose post-purification. Tags can inhibit crystallization^3^, interfere with protein interactions^4^, and produce an unhelpful immune response in vivo^5^. Tags may be removed by proteolysis but this extra step is time-consuming, often inefficient and reduces overall yield^6,7^.

There are already a multitude of affinity tags^1,8–11^. However, each tag presents its own limitations. The most widely used, the His-tag, is not without faults. There are many examples of His-tagging disrupting protein solubility^12^, structure^13,14^, and function^15,16^. His-tag purification faces particular challenges from leakage of metal ions into downstream assays^17,18^ and substantial immunogenicity^5^. The four-amino acid C-tag is less immunogenic but is only functional at the C-terminus^8^. Apart from purification, it would be desirable to use peptide tags for assembly or immobilization, but the low stability of peptide interactions is frequently limiting^19,20^.

**Fig. 1:**
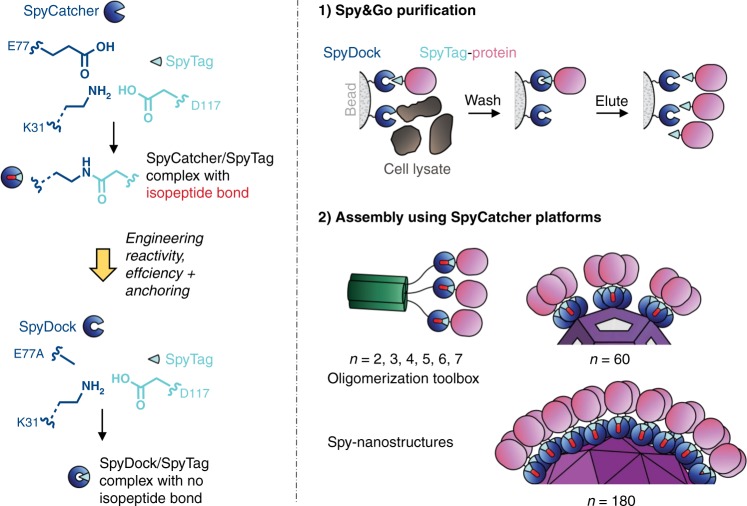
Overview of Spy&Go. SpyTag/SpyCatcher interact irreversibly by spontaneous isopeptide bond formation, promoted by E77. SpyTag purification requires the generation of reversible interaction with SpyDock in 3 steps: blocking reaction, enhancing efficiency of SpyTag binding and precise immobilization on resin. Spy&Go then enables purification of SpyTag-fusions, setting the stage for modular oligomerization or multimerization

We previously developed a peptide-protein pair, SpyTag and SpyCatcher, that spontaneously forms a covalently-linked complex^21,22^ (Fig. 1). We continued the progress by increasing the rate of reaction via the evolved SpyTag002 and SpyCatcher002 versions^23^. SpyTag technology has enabled diverse applications including Plug-and-Display vaccine assembly^24–26^, multivalent activation of signaling^27^, modular antibody decoration^28^, and living or catalytic biomaterials^29–32^. Up until now, Spy proteins were nearly always purified via a His-tag, which required further tag cleavage for immunological applications^24^. Here, we report the development of an affinity chromatography technique employing SpyTag as a purification tag, by step-wise engineering of the SpyCatcher protein partner (Fig. 1). In addition, to extend the modular expansion of protein function, we establish a toolbox for oligomerization of proteins of interest using SpyTag-mediated covalent reaction. These oligomers shed light on the nature of multivalent signaling of DR5 to induce cancer cell killing.

## Results

### Establishment of the Spy&Go purification system

**Fig. 2:**
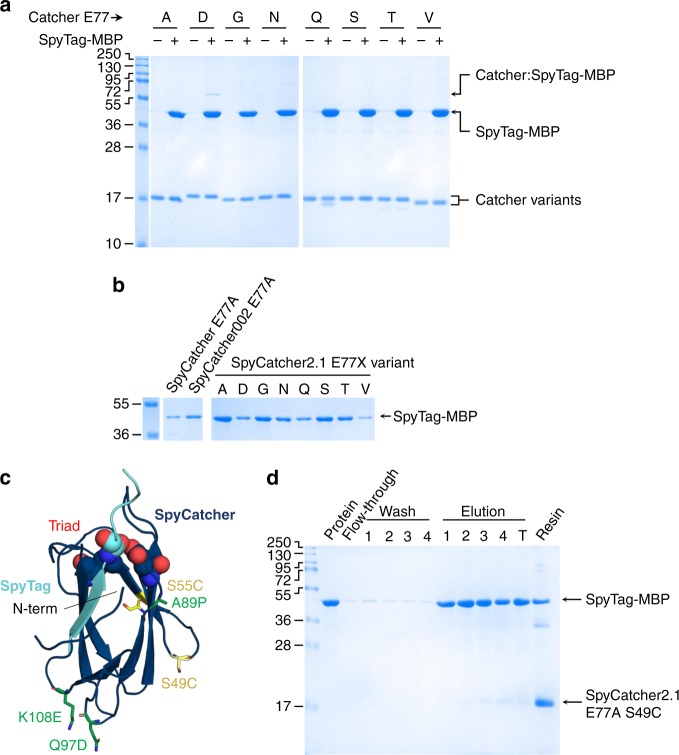
Design of SpyDock. **a** All small residues except D blocked SpyCatcher reaction. E77 was mutated to the indicated residue and incubated with SpyTag-MBP for 24 h, before boiling in SDS and analysis by SDS-PAGE with Coomassie staining. **b** Additional mutations in SpyCatcher enhanced Spy&Go purification. The original SpyCatcher, the evolved SpyCatcher002 or SpyCatcher2.1 bearing the E77A mutation were compared for capture and elution of SpyTag-MBP (SDS-PAGE with Coomassie staining). Further E77X mutations were similarly explored on SpyCatcher2.1. **c** Positions of mutations screened for SpyDock acceleration, based on Protein Data Bank 4MLI; CnaB2 triad represented in spheres, anchoring sites in yellow, and SpyCatcher2.1 accelerating mutations in green. **d** S49C resin anchoring allowed efficient SpyDock purification. SpyCatcher2.1 E77A S49C was coupled to SulfoLink resin and tested for SpyTag-MBP capture and release (SDS-PAGE with Coomassie staining)

As a first step to establish Spy&Go purification, the formation of an isopeptide bond between SpyCatcher and SpyTag must be abrogated, to make possible the elution of SpyTag-fusions. To generate a non-reactive “pseudo-SpyCatcher”, the activating glutamic acid residue in the CnaB2 triad (E77, Fig. 1) was mutated to aspartic acid to retain the charge, or to alanine, glycine, asparagine, glutamine, serine, threonine, or valine to remove any possibility of proton donation/acceptance^33^. We mixed SpyTag-MBP and the E77X variants at 25 °C for 24 h. E77D still showed a small amount of reaction with SpyTag-MBP, but no trace of reaction was seen with any other mutation (Fig. 2a).

To advance Spy&Go purification further, we hypothesized that the rate of association between SpyTag and the SpyCatcher moiety could be limiting. We have previously generated SpyCatcher002 through phage display evolution for enhanced interaction with SpyTag^23^. Here we additionally increased the negative surface charge on SpyCatcher and reduced the flexibility in one of the loops through proline substitution, generating SpyCatcher2.1. When purified SpyTag-MBP was incubated with the different E77X variants, SpyCatcher002 led to more efficient capture and elution than SpyCatcher, while SpyCatcher2.1 was more effective still (Fig. 2b). Comparing alternative E77 mutations for SpyCatcher2.1, E77A led to recovery of the highest amount of SpyTag-MBP (Fig. 2b). Hence, this mutant was taken forward for subsequent development.

We aimed to attach SpyCatcher2.1 E77A site-specifically to sepharose beads, for maximum accessibility to SpyTag-fusions. A unique cysteine was introduced in SpyCatcher2.1 E77A at three positions. Cysteine introduction sites were selected for efficient coupling to iodoacetyl-activated (SulfoLink) beads as well as for minimal disruption to SpyTag/SpyCatcher2.1 binding, by having the cysteine substitutions distal from the CnaB2 reactive triad (Fig. 2c). Three mutations were examined: N-terminal cysteine (N-term Cys) preceding the coding sequence of SpyCatcher2.1, S49C and S55C^34,35^ (Fig. 2c). After immobilizing SpyCatcher2.1 E77A with each cysteine mutation, purified SpyTag-MBP was mixed with the resin, washed, and eluted. All Cys mutants showed similar SpyTag-MBP retention and minimal leak-through during washes, but S49C had the least SpyTag-MBP left on the resin after 4 elutions (Fig. 2d and Supplementary Fig. 1). Therefore, the finalized protein for Spy&Go purification was SpyCatcher2.1 S49C E77A, hereafter termed SpyDock (amino acid sequence in Supplementary Fig. 2). SpyDock can be coupled to SulfoLink resin to achieve 14.2 mg of SpyDock protein per mL of resin (Supplementary Fig. 3).

**Fig. 3:**
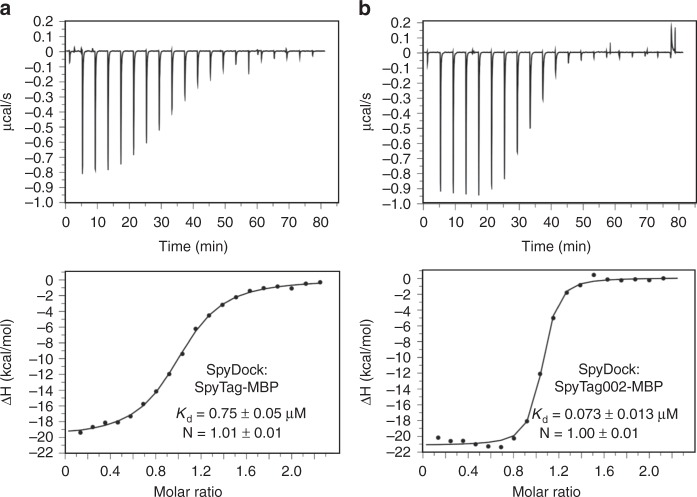
Affinity of SpyDock for its targets. ITC binding isotherms for SpyDock binding at 25 °C to **a** SpyTag-MBP or **b** SpyTag002-MBP. Error bars represent the uncertainty in fit to the binding curve using a 1:1 binding model

To test how well SpyDock bound to SpyTagged proteins, we used isothermal titration calorimetry (ITC) to measure the dissociation constant (*K*_d_) for SpyDock’s interaction with SpyTag-MBP (Fig. 3a) or SpyTag002-MBP (Fig. 3b). Both proteins bound with 1:1 stoichiometry. SpyTag002-MBP (*K*_d_ = 0.073 μM) bound ~10-fold tighter than SpyTag-MBP (*K*_d_ = 0.75 μM), consistent with the improved properties of the second-generation Tag/Catcher technology^23^.

### Purification of model proteins

**Fig. 4:**
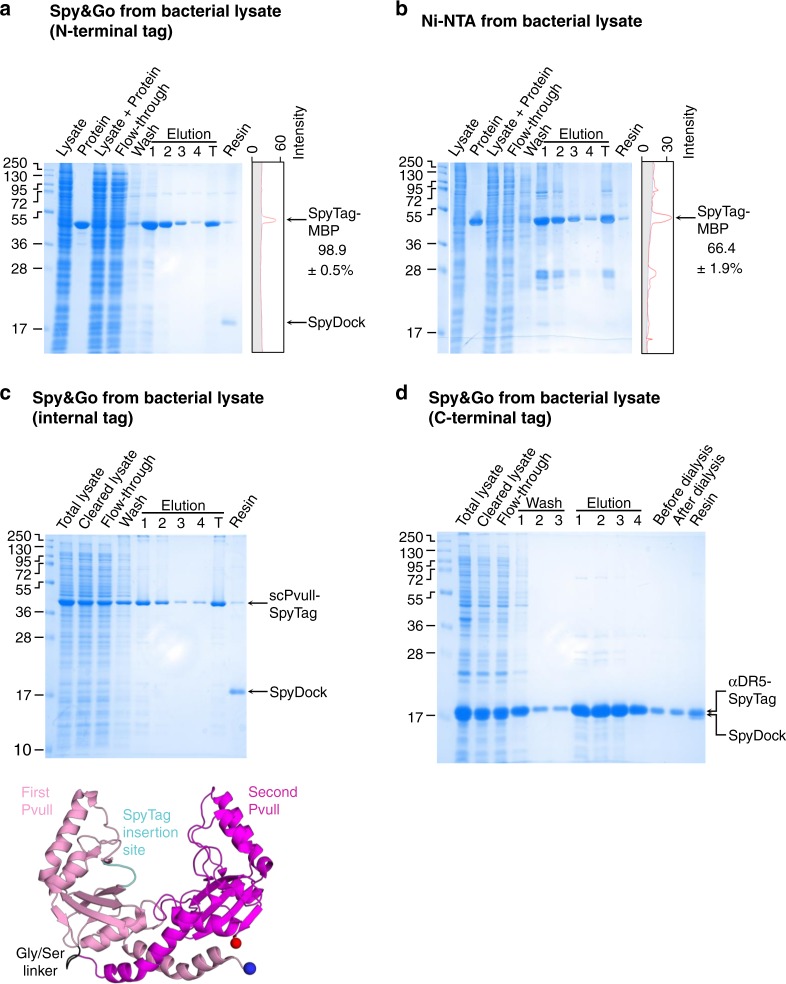
Spy&Go from bacterial expression. **a** SpyTag-MBP was purified from *E. coli* clarified lysate by Spy&Go. Protein: input SpyTag-MBP protein. T: total pooled elutions. Purity of T was determined by densitometry (right); gray represents background lane intensity (mean ± 1 s.d., *n* = 3). **b** Ni-NTA purification of SpyTag-MBP from the same lysate via its His-tag. **c** Spy&Go purification of scPvuII-SpyTag (SpyTag at an internal loop, shown schematically) from bacterial lysate. **d** Spy&Go purification of the nanobody αDR5-SpyTag (C-terminal fusion) from bacterial lysate. All fractions were analyzed by SDS-PAGE with Coomassie staining

With the Spy&Go resin finalized, we sought to evaluate the capability of the system for purifying proteins from cellular material. Affinity purification becomes easier when proteins are highly over-expressed, so we challenged the system by doping a low amount of purified SpyTag-MBP into clarified *E. coli* lysate at 0.36 mg protein per g of wet cell weight. As a comparison, we typically obtain ~5 mg of purified recombinant protein per g of *E. coli* wet cell weight^26^. A series of optimizations established that 2.5 M imidazole at neutral pH was efficient at eluting SpyTag from SpyDock, with lower imidazole concentrations used in the wash buffer^36^. Imidazole is an inexpensive and highly soluble reagent, already present in most biochemistry laboratories. As a bench mark for Spy&Go purification, the SpyTag protein also had a His_6_-tag, so that the same protein and lysate could be compared for the common approach of Ni-NTA purification. The same amount of His-tagged SpyTag-MBP in clarified *E. coli* lysate was mixed with Ni-NTA resin at the same volume. Spy&Go-based purification enabled purification of SpyTag-MBP with higher purity (98.9 ± 0.5%) than via Ni-NTA purification (66.4 ± 1.9%), as seen in the pooled elution fractions measured by gel densitometry (Fig. 4a, b). The ability of the resin to bind and sequester a low concentration of input protein also indicates the sensitivity of Spy&Go as a purification platform.

SpyTag is the most widely used partner of SpyCatcher in the literature^22,37,38^, so our purification approaches focused on this tag version. However, we also validated that Spy&Go was efficient for purification of a SpyTag002-fusion^23^ (Supplementary Fig. 4A).

Additionally, Spy&Go resin was still capable of purifying SpyTag-MBP from bacterial lysate after storage of resin in 20% (v/v) ethanol for 12 weeks. Therefore Spy&Go resin showed good stability, as long as microbial growth was inhibited (Supplementary Fig. 4B).

### Spy&Go allowed purification with SpyTag at different sites

Some affinity tags can only be placed at either the N- or C-terminus, thus restricting experimental flexibility, especially if a functional site of the protein of interest is close to a terminus^1,8,39^. To test capture of SpyTag inserted in the loop of a protein, we generated a single-chain dimer of the restriction enzyme PvuII. scPvuII-SpyTag was purified efficiently from soluble *E. coli* expression using Spy&Go (Fig. 4c). To test capture of SpyTag at the C-terminus of a protein, we expressed a nanobody to Death Receptor 5 (DR5) (αDR5-SpyTag) in the cytosol of *E. coli* and showed efficient purification using Spy&Go (Fig. 4d). A single round of purification is not expected to achieve 100% purity and proteins may be subsequently polished using standard methods such as size-exclusion chromatography or ion-exchange chromatography, as may assist subsequent applications.

To test purification of a different class of protein, the enhanced green fluorescent protein mClover3 was genetically fused with SpyTag at its N-terminus and expressed solubly in *E. coli*. After purification and dialysis, the fluorescence of mClover3 was comparable to the same protein purified via its His-tag using Ni-NTA (Supplementary Fig. 5), supporting that Spy&Go purification maintained the functionality of purified proteins. From lysate purifications, the resin capacity ranged from 4–13 mg of protein per mL of resin, depending on the location of the SpyTag and protein used.

We established effective regeneration of Spy&Go resin via stripping using 4 M imidazole, guanidinium hydrochloride, and NaOH (Supplementary Fig. 6A). Following regeneration of the resin, the smaller protein αDR5-SpyTag was purified by Spy&Go, while SpyTag-MBP (previously purified using the same resin batch) was not detected (Supplementary Fig. 6B). Purification of αDR5-SpyTag using new resin or regenerated resin gave similar results (Supplementary Fig. 6B).

### Spy&Go allowed purification from mammalian expression

**Fig. 5:**
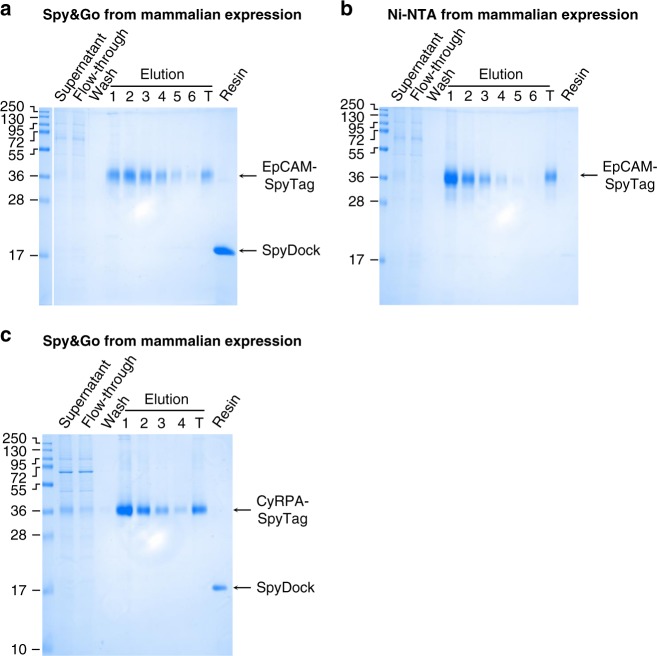
Spy&Go from mammalian expression. **a** HEK293T cells were transfected with the extracellular region of EpCAM fused to SpyTag and a His-tag (EpCAM-SpyTag). EpCAM-SpyTag was purified from the clarified cell supernatant using Spy&Go. Fractions were analyzed by SDS-PAGE with Coomassie staining. T: total pooled elutions. Resin: resin post-elution. **b** Ni-NTA purification of EpCAM-SpyTag as in **a**. **c** Spy&Go purification of CyRPA-SpyTag from Expi293HEK cells as in **a**

Some affinity tags do not work well in particular cell expression systems, requiring extra materials or steps^40^. Transient mammalian expression is becoming a dominant route for the production of many therapeutic and research proteins because of the rapid pipeline, high degree of folding quality control, and the native post-translational modifications (notably N-linked glycosylation)^41^. Epithelial cell adhesion molecule (EpCAM) is a widely used marker for capture of circulating tumor cells and its adhesive interactions may affect metastasis^42,43^. We cloned the soluble extracellular region of EpCAM with SpyTag and His-tag at the C-terminus and expressed this glycoprotein through transient transfection in HEK293T cells. After expression, the same volume of supernatant from the cell culture was incubated with Spy&Go resin or Ni-NTA resin. EpCAM-SpyTag was efficiently purified using Spy&Go, as with Ni-NTA (Fig. 5a, b). The heterogeneous gel mobility of EpCAM-SpyTag is expected because of various glycoforms being secreted.

We previously expressed constructs with a His-tag, to allow bench-marking of our purification, but we also purified Cysteine-rich Protective Antigen (CyRPA) with a SpyTag but no His-tag. CyRPA from *Plasmodium falciparum* is a promising blood-stage antigen for malaria vaccination^44^. CyRPA-SpyTag was transiently expressed in Expi293HEK cells and efficiently purified by Spy&Go (Fig. 5c). Overall, we have shown that Spy&Go is a viable platform for the purification of proteins with SpyTag at the N-terminus, C-terminus or an internal site from either bacterial or mammalian expression systems.

### Oligomeric assembly of SpyTag-fusions

**Fig. 6:**
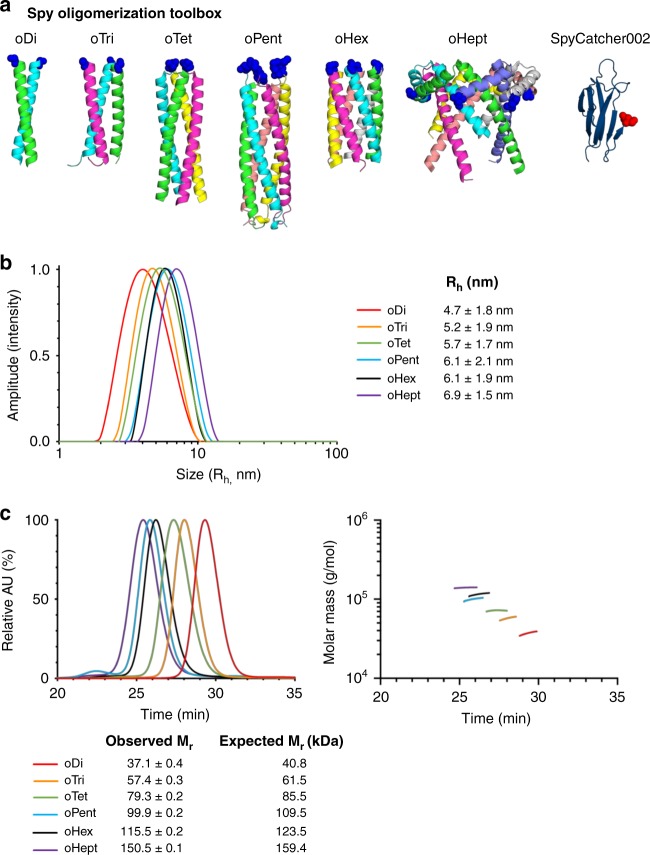
Construction of oligomeric toolbox. **a** Spy Oligomerization Toolbox from establishing a panel of coiled coils with valency from 2 to 7 for fusion to SpyCatcher002. The coiled coils are presented with each color representing one chain and the N-terminus colored as a blue ball. The C-terminus of SpyCatcher002, where it will be linked to the coiled coil, is colored as a red ball. **b** DLS to show assembly of each SpyCatcher002-oligomer, with hydrodynamic radius (*R*_h_) (mean ± 1 s.d., *n* = 10) for each assembly. **c** SEC-MALS to show assembly of each SpyCatcher002-oligomer. The peak shows the normalized absorbance units (AU) at 280 nm of the SpyCatcher002-oligomer species from SEC. The horizontal line shows the distribution of molar mass (g/mol) of the species in the peak from MALS. Expected and observed molecular weight (*M*_r_) is shown alongside, with error bars representing the uncertainty in fit to the molar mass curve

An important feature of using SpyTag as the peptide tag for a protein of interest is that SpyTag can then enable a range of subsequent bioconjugation reactions. For example, SpyTag allows irreversible immobilization on surfaces or hydrogels^29,30,45^, or high-level multimerization on virus-like particles to accelerate vaccine generation^24,26,37^. To extend the application of SpyTag-fusions, here we also explored the ability for rapid assembly of dimers, trimers, tetramers etc. of the protein of interest. We aimed to prepare a panel of parallel coiled coils fused to SpyCatcher002 that could spontaneously self-assemble into different homo-oligomers with valency from 2–7 (Fig. 6a). oDi would represent a dimeric oligomer and oTri a trimeric oligomer and so on.

For oDi, oTri and oHex, we selected coiled coils that were previously computationally designed by the Woolfson laboratory, based on knowledge of key side-chain interactions determining valency and stability, and validated after solid-phase peptide synthesis^46,47^. We increased the repeat number to favor high stability^48^, genetically fused the coiled coils to SpyCatcher002, and expressed constructs in the cytosol of *E. coli*. The best oTet was the coiled coil from VASP (vasodilator-stimulated phosphoprotein)^49^. For oPent, we selected the coiled coil from COMP (cartilage oligomeric matrix protein)^50^. Finally for oHept, we tested the coiled coil from IMX313, originally adapted from complement C4 binding protein^51^. These platforms, containing a His-tag, were purified in good yield after Ni-NTA and gel-filtration chromatography. We validated the composition of each of the coiled coil subunits by electrospray ionization mass spectrometry (Supplementary Fig. 7).

The purified SpyCatcher002-oligomers were subjected to dynamic light scattering (DLS) to verify monodispersity and correct assembly. DLS indicated increasing valency from oDi to oHept based on the increasing hydrodynamic radius (R_h_) (Fig. 6b). We further analyzed the oligomers by size-exclusion chromatography/multi-angle light scattering (SEC-MALS). SEC-MALS also analyzes uniformity but additionally characterizes the molecular weight of the assembly. The observed molecular weights were within 10% of the expected assembled molecular weights (Fig. 6c), suggesting successful assembly by each oligomer from oDi up to oHept.

In our hands, the previously designed tetrameric coiled coil, CC-Tet^46^, when fused to SpyCatcher002, had an observed mass that deviated substantially from the expected mass (Supplementary Fig. 8). We also found that SpyCatcher002 fused to the designed CC-Hept^47^ gave satisfactory assembly (Supplementary Fig. 8), but expressed poorly and was prone to aggregate. Therefore the final Spy oligomerization toolbox was a mixture of computationally designed or natural coiled coils (amino acid sequences shown in Supplementary Fig. 9).

### Oligomeric assembly of a nanobody

DR5 is a pro-apoptotic receptor overexpressed on many cancer cells. DR5 activation promotes cell death in response to binding of TNF-related apoptosis-inducing ligand (TRAIL)^52^. DR5 targeting shows promise as a target for cancer therapy^53^. There has been extensive interest in the clustering of DR5^53–60^, because dimeric anti-DR5 IgG typically causes weak activation of DR5 signaling^53^. Previous attempts at clustering focused on linear chains of anti-DR5 agonists. We explored the use of multivalent display of an agonist nanobody (αDR5), to evaluate the effect of increasing nanobody valency on triggering cancer cell death. Nanobodies are a convenient monomeric scaffold, with the single chain easily expressed in *E. coli*. αDR5 was fused genetically to SpyTag and was purified efficiently via Spy&Go (Fig. 4d). We then mixed this nanobody with the Spy-coiled coil platforms for convenient modular preparation of dimers, trimers etc. up to heptamers.

**Fig. 7:**
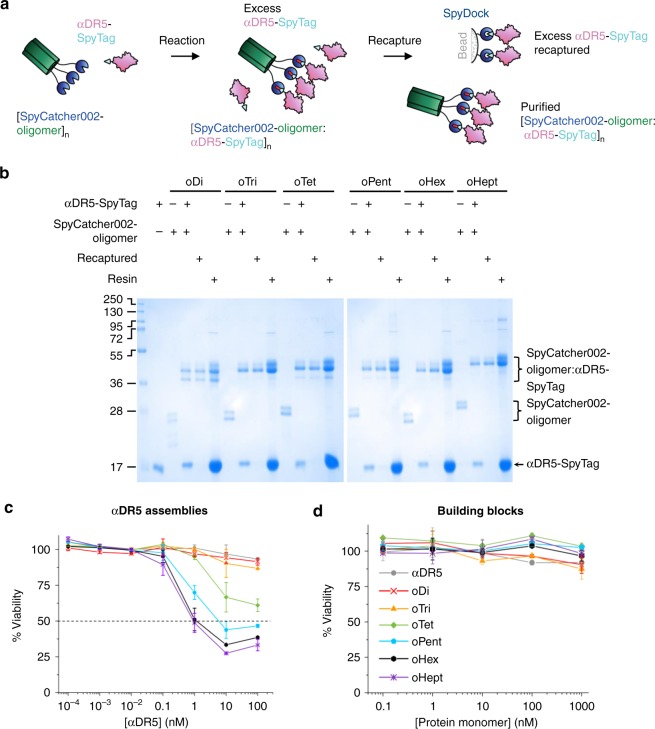
Oligomer panel tested for cancer cell killing. **a** Cartoon depicting depletion of free SpyTag-ligand using Spy&Go resin. **b** Coupling of SpyTag-fusion to coiled coil series. αDR5-SpyTag was incubated with each SpyCatcher002-coiled coil and analyzed by SDS-PAGE with Coomassie staining. In Recaptured lanes, excess αDR5-SpyTag was removed from the coiled coil conjugate by an additional passage through Spy&Go resin. **c** Dose-response curve of MDA-MB-231 cell viability when treated with different concentrations of αDR5 conjugated to each SpyCatcher002-oligomer platforms. The line at 50% cell viability shows the cut-off for EC_50_ calculation. The *x*-axis is normalized to the concentration of αDR5 monomer. Error bars represent mean ±1 s.d., *n* = 2. **d** MDA-MB-231 viability upon incubation as in **c** with the building blocks of αDR5 alone or coiled coils alone. Error bars represent mean ± 1 s.d., *n* = 3

αDR5-SpyTag was incubated with each SpyCatcher002-oligomer and efficient covalent reaction was confirmed by SDS-PAGE. Spy&Go also enabled us to remove excess αDR5-SpyTag from the mixture, by incubating with Spy&Go resin to recapture unconjugated αDR5-SpyTag, leaving only oligomer:nanobody complex in the flow-through (Fig. 7a, b).

### DR5 signal activation by the nanobody oligomeric series

To assess the potency of these oligomeric SpyCatcher002 platforms, we chose a TRAIL-sensitive human breast cancer cell-line MDA-MB-231^54^. We tested a broad range of doses, from 0.1 pM–100 nM for each SpyCatcher002-oligomer:αDR5-SpyTag and compared death induction after 24 h of treatment. While monomeric unconjugated αDR5-SpyTag, dimeric, trimeric, and tetrameric conjugates failed to display any cell killing within this range, we observed a clear stoichiometry-dependent dose-response for higher-order αDR5-conjugates (Fig. 7c). All higher-order conjugates (pentameric, hexameric and heptameric) showed potency at sub-nanomolar concentrations. The pentameric complex resulted in killing of 50% of the cells (EC_50_) at 0.9 nM αDR5. Similarly, the hexamer gave a EC_50_ of 0.42 nM whereas the heptamer had high potency at 0.31 nM EC_50_ (Fig. 7c). As a control, we found that unconjugated SpyCatcher002-oligomer constructs did not elicit any dose-dependent loss of cell viability (Fig. 7d).

## Discussion

We have established a system for purification of SpyTag-fused proteins using an immobilized unreactive SpyCatcher variant. The engineering of SpyDock enables efficient binding of SpyTag, facilitating capture and elution of the protein of interest for the different SpyTag iterations. His-tag/Ni-NTA purification has been optimized over decades but our initial results suggest favorable comparison to Spy&Go for purification from bacteria. We also demonstrated that mammalian proteins can be efficiently purified using SpyTag. As anticipated from the flexible location of SpyTag for covalent reaction with SpyCatcher^21,37,38,45,61^, SpyTag can be located at either terminus of the protein of interest or at an internal site for Spy&Go purification. We have also shown that Spy&Go resin can be effectively regenerated and retains its function after storage for months. The current elution conditions for Spy&Go are unconventional (2.5 M imidazole in Tris-phosphate buffer). Nevertheless, we have observed little aggregation and good yields for the different classes of proteins so far explored. After dialyzing away imidazole, we validated biological activity for mClover3 fluorescence and DR5 agonist cellular activation. We previously demonstrated the tolerance of various enzymes and fluorescent proteins to similar high imidazole concentrations^36^.

Beyond primary purification, Spy&Go also facilitates the generation of purified nanoassembly preparations. We could remove the excess αDR5-SpyTag from each conjugated SpyCatcher002-oligomer:αDR5-SpyTag complex by incubating the mixture with Spy&Go resin. Since SpyTag reacts irreversibly with SpyCatcher002, the reconstitution reaction renders the conjugated SpyTag unavailable for binding to SpyDock.

Modularity is a defining principle in accelerating biological research, moving from painstaking case-by-case artisanal optimization to an efficient and automatable assembly-line^62^. Previous coiled coils were already individually explored as genetic fusion partners for proteins^25,63–65^, however, optimizations would be needed for each distinct protein^24,37,65^. Examples include heterogeneous populations of 5–7 oligomers instead of a monodisperse heptamer^66^, failed assembly of a trimer-fusion protein^67^, and severe degradation of dimers in *E. coli* cytosol^68,69^.

The characterized Spy-based oligomerization toolbox can alleviate this problem, separating the expression/folding/post-translational modification of coiled coils and cargo protein before uniting via SpyTag/SpyCatcher. Spy&Go-purified αDR5-SpyTag readily reacted to completion with multivalent SpyCatcher002-oligomer to form a panel of valency-defined platforms to understand the effect of higher valencies on cellular signaling and the downstream apoptotic effect. Up until now, most multimeric anti-DR5 or soluble TRAIL constructs consist of a linear arrangement linked by simple Gly-Ser linkers extending to 5 repeats^55^, 6 repeats^56,57^ and in one case 8 repeats^57^ of the agonist against DR5^53^. However, the formation of a linear complex may not mimic the clustering by the native TRAIL to DR5 receptor. A “combody” comprising a genetic fusion of the coiled coil COMP with an agonistic αDR5 was previously made^70^, but direct fusion may not be optimized for other proteins or coiled coil combinations, and no higher valencies were reported. We note that we have characterized SpyCatcher002-coiled coil valency by DLS and SEC-MALS at micromolar concentrations. Both natural and synthetic multimers may start to dissociate as one approaches low nanomolar or picomolar concentrations, where cells may still respond to DR5 clustering but bulk biophysical assays become very difficult^46,47^. In future work, single-molecule assays may be the best way to understand molecular and cellular behavior in this low concentration regime^71,72^.

We envision that the oligomerizing SpyCatcher002-coiled coil platforms can advance the study of valency-dependence on diverse cellular signaling processes^73,74^. By combining Spy&Go purification with coiled coil nanoassembly, SpyTagging may help to accelerate the exploration and exploitation of protein space.

## Methods

### Plasmids and cloning

Constructs were cloned by standard PCR methods and assembled using Gibson assembly. Inserts were verified by Sanger sequencing. pDEST14-SpyCatcher2.1 was derived from pDEST14-SpyCatcher002^23^ (GenBank MF974388 and Addgene plasmid ID 102827) with additional A89P, Q97D and K108E mutations (see below). pDEST14-SpyCatcher2.1 S49C E77A (SpyDock) (Supplementary Fig. 2, GenBank MK637462, Addgene plasmid ID 124618) has the organization: His_6_, SpyCatcher2.1 with E77A S49C mutations, GSSGS. pDEST14-SpyCatcher2.1 E77X S49C has the same organization as SpyDock except with E77X mutations instead, with X being D, G, N, Q, S, T, or V. pDEST14-SpyCatcher2.1 E77A N-term Cys has the same organization as SpyDock except with a cysteine-anchoring mutation at the 6^th^ amino acid residue preceding SpyCatcher2.1 E77A instead of S49C. pDEST14-SpyCatcher2.1 S55C E77A has the same organization as SpyDock except with a S55C mutation instead of S49C mutation. pET28a-SpyTag-MBP^21^ (Addgene plasmid ID 35050) and pET28a-SpyTag002-MBP^23^ (GenBank MF974389 and Addgene plasmid ID 102831) were as described. pENTR4-EpCAM-SpyTag (GenBank MK637463) was derived from pENTR4-EpCAM-SnoopTagJr^30^ (GenBank MH511516) with the organization: tissue plasminogen activator (tPA) secretion leader sequence, extracellular domain of human EpCAM protein (EpCAM; residue 24–265), (GSG)_2_, SpyTag, GEGS, His_6_. pENTR4-LPTOS-CyRPA-SpyTag^26^ (GenBank MH425516) was previously published. pET28a-αDR5-SpyTag (GenBank MK637464) was derived from pET28a-SnoopTag-αDR5-SpyTag (GenBank KU500643)^54^ with the organization: αDR5 (4E6 nanobody^55^), (GGGGS)_2_, SpyTag. pET28a-SpyTag-mClover3 has the organization: SpyTag, SGGGSG, mClover3^75^, GSGSGS, His_6_. pET28a-scPvuII-SpyTag (GenBank MK637465) has the organization: His_6_, SSG, PvuII from *Proteus vulgaris*, GSG, TEV cleavage site, GGSG, SpyTag, GSGG, PvuII. SpyTag along with surrounding spacers was inserted into a loop of the first PvuII between amino acid residues 75 and 76. PvuII constructs had the D58A mutation to block DNA cleavage. pDEST14-SpyCatcher002 was linked to coiled coil inserts consisting of oDi^46^ (GenBank MK637466, Addgene plasmid ID 124661), oTri^46^ (GenBank MK637467, Addgene plasmid ID 124662), oTet^49^ (GenBank MK637468, Addgene plasmid ID 124663), oPent^50^ (GenBank MK637469, Addgene plasmid ID 124664), oHex^47^ (GenBank MK637470, Addgene plasmid ID 124670), oHept^51^ (GenBank MK637471, Addgene plasmid ID 124671), CC-Tet^46^ (GenBank MK637472) or CC-Hept^47^ (GenBank MK637473). The constructs have the organization: His_6_, DYDIPTT spacer, TEV cleavage site, SpyCatcher002, GSSGSGSGS, coiled coil inserts, GSGSG, C-tag. Coiled coil DNA was synthesized by IDT DNA Technologies (Supplementary Fig. 9). oDi, oTri, oHex, CC-Tet, and CC-Hept have 5 heptad repeats, instead of 4 repeats reported in the literature, to increase the stability of coiled coil assembly^48^.

### SpyDock rational design

To complement the increase in positively charged residues of SpyTag002 compared to SpyTag, mutations Q97D and K108E were made on SpyCatcher002 to increase the negatively charged residues, making the SpyDock precursor, SpyCatcher2.1, to improve the electrostatic interaction with the positively-charged SpyTag or SpyTag002 (Fig. 2c). Residues Y83 and E85 within the long A79-A89 loop in SpyCatcher make key interactions with residues Y9 and K10 of SpyTag in the SpyTag/SpyCatcher crystal structure^76^. These wild-type residues were also positively selected during directed evolution to produce SpyTag002, supporting the importance of the residues for rapid isopeptide bond formation^23^. Inclusion of prolines in turns and loops has previously been shown to stabilize proteins^36,77–79^. Hence, the A89P mutation was included to stabilize the A79-A89 loop (Fig. 2c). The E77 was targeted to nullify the isopeptide bond formation, with A, D, G, N, Q, S, T, and V mutations as candidates (Fig. 1). These mutations were postulated to enable better binding and retention of SpyTag to SpyDock during the binding and washing steps, along with easier elution.

### Bacterial protein expression

pET28a-SpyTag-MBP, pET28a-SpyTag002-MBP, and pET28a-SpyTag-mClover3 were transformed into chemically competent *E. coli* BL21 (DE3) RIPL (Agilent Technologies). pET28a-scPvuII-SpyTag was transformed into T7 Express *lysY*/*I*^q^ (NEB). pDEST14-SpyCatcher2.1 S49C E77A (SpyDock), pDEST14-SpyCatcher2.1 S49C E77X variants, pDEST14-SpyCatcher2.1 E77A N-term Cys, pDEST14-SpyCatcher2.1 S55C E77A and pDEST14-SpyCatcher002-coiled coil fusions were transformed into chemically-competent *E. coli* C41 (DE3), a kind gift from Anthony Watts (University of Oxford). pET28a-αDR5-SpyTag was transformed into *E. coli* BL21 (DE3) RIPL containing a gene encoding phosphogluconolactonase, to degrade 6-phosphogluconolactone, which promotes protein gluconoylation^80^. The cells were plated on LB agar supplemented with 50 µg/mL kanamycin (pET28a) or 100 µg/mL ampicillin (pDEST14). For αDR5-SpyTag, 34 µg/mL chloramphenicol was added alongside kanamycin throughout. The plates were incubated at 37 °C overnight until colonies were observed.

Single colonies of pET28a-SpyTag-MBP, pET28a-SpyTag002-MBP, pET28a-SpyTag-mClover3, pET28a-scPvuII-SpyTag, and pDEST14-SpyDock, all variants of pDEST14-SpyCatcher2.1 S49C E77X, pDEST14-SpyCatcher2.1 E77A N-term Cys, pDEST14-SpyCatcher2.1 E77A S55C, and all variants of pDEST14-SpyCatcher002-coiled coils were picked and inoculated into 10 mL LB medium supplemented with 50 µg/mL kanamycin (pET28a) or 100 µg/mL ampicillin (pDEST14), incubated at 37 °C with shaking at 200 rpm for 16 h. The cultures were then inoculated into 1 L LB supplemented with 50 µg/mL kanamycin (pET28a) or 100 µg/mL ampicillin (pDEST14) and 0.8% (w/v) glucose (except for pDEST14-SpyCatcher002-coiled coils), incubated at 37 °C with shaking at 200 rpm until *A*_600_ 0.5–0.6, when the cultures were induced with 0.42 mM isopropyl β-d-1-thiogalactopyranoside (IPTG) (Fluorochem). Cultures of pET28a-SpyTag-MBP, pET28a-SpyTag002-MBP, pET28a-SpyTag-mClover3, pET28a-scPvuII-SpyTag, and pDEST14-SpyDock, all variants of pDEST14-SpyCatcher2.1 S49C E77X, pDEST14-SpyCatcher2.1 E77A N-term Cys, and pDEST14-SpyCatcher2.1 E77A S55C were grown further for 4 h with shaking at 200 rpm at 30 °C. Cultures of pDEST14-SpyCatcher002-oDi, pDEST14-SpyCatcher002-oTri, pDEST14-SpyCatcher002-oTet, pDEST14-SpyCatcher002-oPent, and pDEST14-SpyCatcher002-CC-Tet were grown further for 16 h with shaking at 200 rpm at 22 °C. Cultures of pDEST14-SpyCatcher002-oHex, pDEST14-SpyCatcher002-oHept and pDEST14-SpyCatcher002-CC-Hept were grown for 4 h with shaking at 200 rpm at 37 °C.

A single colony of pET28a-αDR5-SpyTag was picked and inoculated into 1 L auto-induction medium (AIMLB0205 from Formedium) supplemented with 50 µg/mL kanamycin and 34 µg/mL chloramphenicol, incubated at 30 °C with shaking at 200 rpm for 24 h. All *E. coli*-expressed proteins were harvested by centrifugation at 4000×*g* for 15 min at 4 °C prior to purification.

### Mammalian protein expression

EpCAM-SpyTag was expressed in adherent HEK293T cells. HEK293T cells were cultured in T175 adhesive culture flasks (Corning) with Dulbecco’s Modified Eagle’s Medium (DMEM) (Sigma-Aldrich) high glucose with 10% (v/v) Fetal Bovine Serum (Sigma-Aldrich), 2 mM l-glutamine, 100 U/mL penicillin, and 100 μg/mL streptomycin (Thermo Fisher Scientific) at 37 °C with 5% CO_2_. Before transfection, the cells were seeded into a T875 5-layer flask (Corning) and upon reaching 50% confluency, they were transferred into serum-free media (DMEM, 2 mM glutamine, 50 U/mL penicillin, 25 mM HEPES added) and mixed with 30 μg pENTR4-EpCAM-SpyTag plasmid per 7.5 mL of media for each flask layer. After 15 min, 2.5 mL media containing 36 μg/mL polyethyleneimine (Sigma-Aldrich) was added to each layer. 10 mL media containing 4.4 μM valproic acid (Sigma-Aldrich), 100 U/mL penicillin, and 100 μg/mL streptomycin was added to each layer 16–20 h later. Cells were then incubated at 37 °C with 5% CO_2_ for another 6 days.

CyRPA-SpyTag was expressed in suspension Expi293HEK cells (Thermo Fisher Scientific). Expi293HEK cells were cultured in Expi293 expression media (Thermo Fisher Scientific) with 50 U/mL penicillin/streptomycin at 37 °C with 7% CO_2_ shaking at 110–125 rpm. Transient transfection of pENTR4-LPTOS-CyRPA-SpyTag was done using the ExpiFectamine 293 transfection kit (Thermo Fisher Scientific). Cells at a density of 2.5 × 10^6^ cells/mL were transfected with 2.7 μL ExpiFectamine 293 Reagent per 1 μg of pENTR4-LPTOS-CyRPA-SpyTag plasmid. After 16–18 h, ExpiFectamine transfection enhancers (Thermo Fisher Scientific) were added and the cell supernatant was harvested 4 days post-transfection.

The cell supernatants were harvested by addition of cOmplete™, Mini, EDTA-free Protease Inhibitor Cocktail (Roche), centrifuged at 1000×*g* for 3 min, and filtered through a 0.45 μm syringe filter to remove cell debris. 2.5% of 10×Ni-NTA or 10×TP buffer (250 mM orthophosphoric acid adjusted to pH 7.0 with Tris base) was added for pH adjustment before affinity chromatography purification.

### Protein purification by Ni-NTA

Purifications were done at 4 °C throughout. All *E. coli*-grown constructs (except αDR5-SpyTag, scPvuII-SpyTag, SpyTag-mClover3, SpyCatcher002-oHex, SpyCatcher002-oHept and SpyCatcher002-CC-Hept) were resuspended in 1×Ni-NTA buffer (50 mM Tris-HCl, 300 mM NaCl pH 7.8; SpyDock along with all variants of SpyCatcher2.1 S49C E77X, SpyCatcher2.1 E77A N-term Cys, SpyCatcher2.1 E77A S55C and SpyCatcher002-oPent had additional 10 mM 2-mercaptoethanol) with cOmplete™, Mini, EDTA-free Protease Inhibitor Cocktail and 1 mM phenylmethylsulfonyl fluoride (PMSF). Cells were lysed by addition of 100 μg/mL lysozyme (Sigma-Aldrich) and 2 U/mL benzonase (Sigma-Aldrich). The lysate was rotated at 25 °C for 30 min and sonicated on ice for 4 × 1 min with 1 min rest period at 50% duty cycle. Clarified cell lysates were centrifuged at 30,000 *g* for 30 min before incubation with Ni-NTA beads (Qiagen) on a rotary shaker for 1 h. The lysate-bead mixture was added onto a polyprep gravity column and washed with 20 packed resin volumes of Ni-NTA wash buffer (10 mM imidazole in Ni-NTA buffer, pH 7.8; SpyDock, all variants of SpyCatcher2.1 S49C E77X, SpyCatcher2.1 E77A N-term Cys, SpyCatcher2.1 E77A S55C and SpyCatcher002-oPent had 10 mM 2-mercaptoethanol for the first 10 packed resin volumes; SpyCatcher002 coiled coil fusions used 75 mM imidazole in Ni-NTA buffer) and eluted with Ni-NTA elution buffer (200 mM imidazole in Ni-NTA buffer, pH 7.8; SpyCatcher002 coiled coil fusions used 350 mM imidazole in Ni-NTA buffer) at 4 °C. Elutions were monitored by *A*_280_ and stopped once *A*_280_ was <1.0. Proteins were dialyzed against 20 mM Tris-HCl pH 8.0 and concentrated, if necessary, using Vivaspin centrifugal concentrator 5 kDa cutoff (GE Healthcare).

SpyDock was further purified on a HiTrap Q HP anion exchange chromatography column (GE Healthcare) connected to an ÄKTA Pure 25 (GE Healthcare) fast protein liquid chromatography (FPLC) system at 4 °C. The protein was eluted with a linear gradient of 0.2–0.35 M NaCl (in 10 mM Tris-HCl pH 8.0 with 1 mM dithiothreitol) at a flow rate of 2 mL/min at 4 °C. Peak fractions were verified by SDS-PAGE gels, dialyzed against 20 mM Tris-HCl pH 8.0, and concentrated using Vivaspin centrifugal concentrator 5 kDa cutoff. Typical yield for SpyDock after Ni-NTA and ionic exchange chromatography is approximately 28 mg/L culture.

All SpyCatcher002 coiled coil fusions were further purified by gel filtration chromatography on a pre-equilibrated HiLoad 16/600 Superdex 200 pg column connected to ÄKTA Pure 25 FPLC system. The mobile phase was 50 mM Tris-HCl, 150 mM NaCl pH 8.0 at a flow-rate of 1 mL/min at 4 °C with *A*_280_ monitored throughout. Fractions were collected corresponding to the size of the oligomeric SpyCatcher002 coiled coil complex: SpyCatcher002-oDi (75–78 mL), SpyCatcher002-oTri (69–72 mL), SpyCatcher002-oTet (66–70 mL), SpyCatcher002-oPent (62–65 mL), SpyCatcher002-oHex (61–65 mL), SpyCatcher002-oHept (56–60 mL). All SpyCatcher002-coiled coil fusions had a yield of approximately 3 mg/L culture, following Ni-NTA and gel filtration chromatography.

### Protein purification by refolding

SpyCatcher002-oHex, SpyCatcher002-oHept, and SpyCatcher002-CC-Hept were found in inclusion bodies and so were purified by refolding. Cells were resuspended in 100 mM Tris-HCl pH 8.0 with cOmplete™, Mini, EDTA-free Protease Inhibitor Cocktail and 1 mM PMSF. Cells were lysed by addition of 100 μg/mL lysozyme and 2 U/mL benzonase and rotated at 25 °C for 30 min. There was one freeze-thaw cycle from −80 °C to 25 °C, followed by addition of 0.5% (v/v) Triton X-100 and sonication on ice for 4 × 1 min with 1 min rest period at 50% duty cycle. Clarified cell lysates were centrifuged at 30,000×*g* for 30 min. The cell pellet was washed with 2 × PBS + 0.1% (v/v) Triton X-100 and then 2 × PBS, with centrifugation at 25,000×*g* for 20 min between steps. The inclusion body pellet was resuspended in 8 M urea in 50 mM Tris-HCl pH 8.0 and refolded by diluting 50-fold into refolding buffer of 0.1 M Tris-HCl pH 8.0, 0.4 M l-arginine and 0.1 M PMSF (for SpyCatcher002-oHept, 5 mM reduced glutathione and 0.5 mM oxidized glutathione were present) for 40 h. The mixture was filtered through a 0.45-μm membrane before incubation with Ni-NTA beads on a rotary shaker for 1 h. The lysate-bead mixture was added onto a polyprep gravity column and washed with 20 resin volumes of Ni-NTA wash buffer (with 75 mM imidazole; SpyCatcher002-oHept had 10 mM 2-mercaptoethanol for the first 10 packed resin volumes) and eluted with Ni-NTA elution buffer (with 350 mM imidazole) at 4 °C. Elutions were monitored by *A*_280_ and stopped once *A*_280_ was <1.0. Proteins were dialyzed against 20 mM Tris-HCl pH 8.0 and concentrated to suitable working concentrations, if needed, using Vivaspin centrifugal concentrator 5 kDa cutoff (GE Healthcare).

### SpyCatcher2.1 E77A variants coupling to SulfoLink resin

Purified SpyCatcher2.1 E77A with various cysteine anchoring residues (N-term Cys, S55C, and S49C) were coupled to SulfoLink Coupling Resin (Thermo Fisher Scientific) according to the manufacturer’s protocol at 25 °C. In short, 20 mg of protein for every 1 mL of resin was reduced by 1 mM tris(2-carboxyethyl)phosphine (TCEP) (Fluorochem, UK) for 30 min, prior to mixing end-over-end with equilibrated resin for 15 min and leaving to stand for 30 min covered by foil. Protein flow-through was aspirated and resin was washed with 10 resin volumes of coupling buffer (50 mM Tris-HCl, 5 mM EDTA, pH 8.5). The resin was blocked with 50 mM L-cysteine-HCl in coupling buffer (MP Biomedicals), mixed end-over-end for 15 min, and left to stand for 30 min. The resin was then washed with 10 resin volumes of 1 M NaCl, and stored in 1xTris-phosphate (TP) buffer (25 mM orthophosphoric acid adjusted to pH 7.0 with Tris base), before adding 0.05% (w/v) NaN_3_ and storing at 4 °C.

### Isothermal titration calorimetry

SpyDock was trapped as a monomer by reduction with 2.5 mM TCEP in 50 mM Tris-HCl with 5 mM EDTA at pH 8.5 and by cysteine modification with 20 mM iodoacetamide in 50 mM Tris-HCl with 5 mM EDTA at pH 8.5 for 30 min in the dark to produce carbamidomethylated SpyDock. Excess TCEP and iodoacetamide were removed by dialysis. SpyTag-MBP, SpyTag002-MBP and the modified SpyDock were subsequently dialyzed twice into 20 mM HEPES pH 7.0 with 150 mM NaCl. Experiments were carried out using a Microcal PEAQ-isothermal titration calorimetry (ITC) calorimeter (Malvern) at 25 °C in 20 mM HEPES pH 7.0 with 150 mM NaCl. 20 μM SpyDock was used in the cell and titrated with 20 injections of 210 μM SpyTag-MBP or SpyTag002-MBP in the syringe. Analyses were carried out using a 1:1 binding model with the MicroCal PEAQ-ITC Analysis software version 1.1.0.1262.

### Purification by Spy&Go

To determine the best cysteine anchoring site, 50 μL packed resin with SpyCatcher2.1 E77A N-term Cys, S55C, or S49C was mixed with 500 μL TP buffer containing 0.09 mg (10× lower than SpyCatcher2.1 E77A available on resin) of Ni-NTA-purified SpyTag-MBP in an Eppendorf tube through batch chromatography. The low amount of SpyTag-MBP introduced was to test the sensitivity of Spy&Go purification. The protein was mixed with the resin for 1 h with tumbling at 4 °C. Standard Spy&Go batch chromatography purification is as follows: the resin was washed 4 × with 10 resin volumes of TP buffer, with incubation at 4 °C shaking at 1,200 rpm for 3 min. Resin was then centrifuged at 4000×*g* for 3 min at 4 °C. The protein was eluted with 4 × 1.5 resin volume of elution buffer ETP (2.5 M imidazole in TP buffer).

For purification of SpyTag-MBP and SpyTag002-MBP from lysate, 0.09 mg of the Ni-NTA-purified proteins were added into ~0.25 g wet cell weight of induced BL21 (DE3) RIPL cleared lysate dissolved in 500 μL TP buffer in an Eppendorf tube containing 50 μL packed Spy&Go resin. The protein-lysate was mixed with the resin for 1 h tumbling at 4 °C. Standard Spy&Go batch chromatography purification was performed to purify the proteins but washed with wash buffer WTP (500 mM imidazole in TP buffer).

EpCAM-SpyTag was purified from the supernatant of HEK293T cells by mixing 50 mL of the supernatant with 0.5 mL packed Spy&Go resin. CyRPA-SpyTag was purified from the supernatant of Expi293HEK cells by mixing 3.5 mL of the supernatant with 50 µL of packed Spy&Go resin. Both were rolling for 1 h at 4 °C. The mixture was then purified by standard gravity column chromatography: resin was washed 4× with 10 resin volumes of TP buffer and eluted with 6 × 1 resin volume of ETP buffer (4 × 1 resin volume for CyRPA-SpyTag). Typical yield for EpCAM-SpyTag after Spy&Go purification was approximately 2.9 mg/L culture and for CyRPA-SpyTag was approximately 8.9 mg/L culture.

For comparison with Ni-NTA resin, the methods described were performed with equivalent volume of packed Ni-NTA resin with the following changes: the cell pellet was resuspended in 50 mM Tris-HCl, 300 mM NaCl pH 7.8, the wash buffer was Ni-NTA wash buffer (30 mM imidazole for SpyTag-MBP, 10 mM imidazole for EpCAM-SpyTag) and elution was carried out with Ni-NTA elution buffer.

For purification of αDR5-SpyTag, the cell pellet from 1 L of culture was centrifuged and resuspended in 10 mL TP buffer with 1 mM dithiothreitol, cOmplete™, Mini, EDTA-free Protease Inhibitor Cocktail and 1 mM PMSF. Cells were lysed by addition of 100 μg/mL lysozyme and 2 U/mL benzonase rotated at 25 °C for 30 min, and subsequently sonicated on ice for 4 × 1 min at 50% duty cycle and spun down at 30,000 *g* to remove cell debris. The cleared lysate was mixed with 1 mL of Spy&Go resin rolling for 1 h at 4 °C. Standard gravity column chromatography purification was performed to purify αDR5-SpyTag with washes using WTP buffer.

For purification of scPvuII-SpyTag and SpyTag-mClover3, the cell pellet from 0.25 L of culture was lysed with 5 mL BugBuster reagent per gram of wet cell weight in the presence of 100 µg/mL lysozyme and 2 U/mL benzonase. The sample was incubated standing at room temperature for 20 min. After spinning down at 30,000×*g* to remove cell debris, the cleared lysate was mixed with Spy&Go resin with rolling for 1 h at 4 °C. Standard gravity column chromatography purification was performed using WTP buffer for washes and 4 × 1 resin volume for elution.

### SpyTag-protein and SpyCatcher reconstitution reaction

To investigate any formation of covalent bond, 5 μM SpyCatcher2.1 S49C with E77X (X being A, D, G, N, Q, S, T, or V) was reacted with 2 × molar excess of SpyTag-MBP (10 μM) at 25 °C for 24 h in TP buffer. Reaction was quenched with SDS-PAGE loading buffer [0.23 M Tris HCl pH 6.8, 24% (v/v) glycerol, 120 μM bromophenol blue, 0.23 M SDS, 100 mM 2-mercaptoethanol] and heating at 95 °C for 5 min in a Bio-Rad C1000 thermal cycler.

To form SpyCatcher002-oligomer:αDR5-SpyTag, 100 μM monomeric concentration of SpyCatcher002-oligomer was mixed with 300 μM αDR5-SpyTag in 200 μL total volume overnight at 25 °C in 50 mM Tris-HCl, 150 mM NaCl, pH 8.0. Excess αDR5-SpyTag was removed by recapturing using Spy&Go resin by incubating the reaction mixture with 50 μL Spy&Go resin for 2 h at room temperature with mixing end-over-end. The mixture was then loaded onto a Micro Bio-Spin column (Bio-Rad) and the conjugated SpyCatcher002-oligomer:αDR5-SpyTag was recovered by centrifugation at 300 *g* for 1 min at 4 °C into a microcentrifuge tube. This process was repeated once. The protein concentrations were measured by Proteoquant BCA assay (Expedeon).

### SDS-PAGE and protein purity quantification

SDS-PAGE was performed using 16% Tris-glycine gels in an XCell SureLock system (Thermo Fisher Scientific). Samples were loaded with final concentration of 1x SDS-PAGE loading buffer. For reduced samples, 100 mM 2-mercaptoethanol was added. SDS-PAGE gels were run at 190 V in 25 mM Tris-HCl, 192 mM glycine, 0.1% (w/v) SDS, pH 8.5. Gels were stained with InstantBlue Coomassie stain (Expedeon), destained with MilliQ water, and imaged using ChemiDoc XRS imager and analyzed with ImageLab (version 6.0.1) (Bio-Rad). In ImageLab, low sensitivity band detection in the final eluted lane (T) was calculated and compared with the protein control lane (Protein) at background subtraction of disk size 2 mm. Percentage purity is defined as 100 × [target protein Band % in lane T/target protein Band % in lane Protein].

### Regeneration of Spy&Go resin

Lysate from bacterial expression of SpyTag-MBP was purified using 0.5 mL Spy&Go resin as above. The resin was equilibrated with 2 × 10 packed resin volumes of TP buffer before regeneration. Spy&Go resin was then regenerated at 25 °C by incubating with 3 × 10 packed resin volumes of 4 M imidazole in TP buffer pH 7.0 for 5 min each time, washing with 1 × TP buffer, incubating with 3 × 10 packed resin volumes of 6 M guanidine hydrochloride pH 2.0 for 5 min each time, washing with 1 × TP buffer, incubating with 3 × 10 packed resin volumes of 0.1 M NaOH for 1 min each time, and washing with 2 × TP buffer before storage in 20% (v/v) ethanol in MilliQ water at 4 °C. Supernatant from each stage was analyzed by SDS-PAGE with Coomassie staining. New resin, resin pre-regeneration and regenerated resin were also analyzed by boiling in SDS-PAGE loading buffer, followed by SDS-PAGE with Coomassie staining. Lysate from bacterial expression of αDR5-SpyTag was added to new resin or regenerated resin and purified using the standard Spy&Go protocol, before analysis by SDS-PAGE with Coomassie staining. To test capture after storage, Spy&Go resin was stored in 20% (v/v) ethanol for 12 weeks at at 4 °C, before purification of SpyTag-MBP from bacterial lysate following the standard protocol.

### Mass spectrometry

An Agilent RapidFire 365 platform, coupled to an Agilent 6550 Accurate-Mass Quadrupole Time-of-Flight (Q-TOF) mass spectrometer, was used to perform intact protein mass spectrometry in positive ion mode with a jet-stream electrospray ion source (Agilent). Proteins with disulfide bonds were reduced with 2.5 mM TCEP in 50 mM Tris-HCl with 5 mM EDTA at pH 8.5 for 1 h before treatment with formic acid. Protein samples at 10 μM in 50 μL volume were prepared on a 384-well polypropylene plate (Greiner) and acidified to 1% (v/v) formic acid. Samples were aspirated under vacuum for 0.4 s on the RapidFire sampling platform and loaded onto a C4 solid-phase extraction cartridge. Following washes with 0.1% (v/v) formic acid at 1.5 mL/min flow-rate for 5.5 s, the samples were eluted to the mass spectrometer with deionized water containing 85% (v/v) acetonitrile and 0.1% (v/v) formic acid at 1.25 mL/min for 5.5 s. The cartridge was equilibrated with deionized water for 0.5 s. Nitrogen drying gas for the ionization source was operated at 13 L/min at 225 °C, with the jet stream sheath gas at a flow-rate of 12 L/min at 350 °C and the nozzle voltage at 1500 V. Data analysis was done using Mass Hunter Qualitative Analysis software version 7.0, with the protein ionization data deconvoluted using the maximum entropy algorithm. Predicted mass was calculated using ExPASy ProtParam, based on all disulfide bonds being reduced and cleavage of the N-terminal formylmethionine.

### SEC-MALS

Samples were prepared at 2 mg/mL in 100 μL in 50 mM Tris-HCl, 150 mM NaCl pH 8.0 and injected into a Superdex 200 HR 10/30 column (GE Healthcare) at 25 °C at a flow-rate of 0.5 mL/min connected to a Shimadzu HPLC system comprising LC-20AD pump, SIL-20AC autosampler and SPD20A UV/Vis detector with 50 mM Tris-HCl, 150 mM NaCl pH 8.0 as running buffer. Light scattering was detected by a Wyatt Dawn HELEOS-II 8-angle light scattering detector and Wyatt Optilab rEX refractive index monitor. The resulting light scattering, refractive index and UV traces were processed in ASTRA 6 (Wyatt Technologies).

### Dynamic light scattering

Samples were prepared at 1 mg/mL in 50 mM Tris-HCl, 150 mM NaCl pH 8.0 and centrifuged for 30 min at 16,900×*g* at 4 °C to remove any aggregates. 20 μL of each sample was loaded into a reusable quartz cuvette and measurements were taken at 20 °C using an Omnisizer (Viscotek) with 10 scans of 10 s each. Data were analyzed using OmniSIZE 3.0 and the intensity distribution from the scans were plotted in Excel.

### Fluorescence assay

A concentration gradient of SpyTag-mClover3 in PBS, purified by either Spy&Go or Ni-NTA, was prepared in triplicate from 15 μM to 0.47 μM in a black, flat-bottom half-area 96-well plate (Corning). The protein was excited at *λ*_ex = _482 ± 16 nm and fluorescence intensity was detected at *λ*_em_ = 530 ± 40 nm, at 40 flashes per well using a CLARIOstar plate reader (BMG Labtech) at 30 °C. The fluorescence intensity was plotted using Microsoft Excel.

### Cell killing assay

MDA-MB-231 cells from American Type Culture Collection were grown at 37 °C  with 5% CO_2_ in Dulbecco’s Modified Eagle Medium (DMEM) (Life Technologies) containing 10% (v/v) Fetal Bovine Serum (Sigma-Aldrich) and 50 U/mL penicillin and 50 μg/mL streptomycin (Sigma-Aldrich). Cells were passaged upon 70–80% confluency and for <3 months in total. MDA-MB-231 cells were seeded into a 96-well plate at 40,000 cells per well in 100 µL DMEM containing 50 U/mL penicillin and 50 μg/mL streptomycin and 1% (v/v) FBS and incubated at 37 °C with 5% CO_2_ for 16 h. Each SpyCatcher002-oligomer:αDR5-SpyTag was prepared at 100 nM monomeric concentration and serial dilutions thereof, in DMEM with antibiotics and 1% (v/v) FBS as above. For a negative control, SpyTag-MBP (at 100 nM and serial dilutions) was added to cells. Cells were washed with sterilized PBS twice before addition of the conjugates. The cells were incubated with the protein samples at 37 °C with 5% CO_2_ for 24 h. Cell viability was tested by addition of 40 µL 0.15 mg/mL Resazurin (Alamar Blue) (Sigma-Aldrich) in PBS at 37 °C with 5% CO_2_ for 4 h and by measuring fluorescence (*λ*_ex_ 544 nm, *λ*_em_ 590 nm) using a SpectraMax3 plate reader with SoftMax Pro 5.4 software (Molecular Devices). The percentage of viable cells was calculated as 100 × (signal of treated cells - signal without cells)/(signal untreated cells–signal without cells). The signal without cells was taken as the resazurin fluorescence in the absence of cells, whereas the signal of untreated cells came from the fluorescence of resazurin with cells that were incubated with DMEM with antibiotics and 1% (v/v) FBS.

### Graphics and sequence analysis

Structures are shown from SpyTag/SpyCatcher (PDB ID 4MLI)^76^, oDi (PDB ID: 4DZM)^46^, oTri (PDB ID: 4DZL)^46^, oTet (PDB ID: 1USE)^49^, oPent (PDB ID: 1VDF)^50^, oHex (PDB ID: 4PN9)^47^, oHept (2YF2)^51^, CC-Tet (3R4A)^46^, CC-Hept (4PNA)^47^, or PvuII (3KSK). PyMOL 2.0 was used to visualize the structure of proteins based on PDB IDs. Amino acid sequence alignment was done with Clustal Omega.

### Software

Microsoft Excel was used to plot DLS and fluorescence graphs. GraphPad Prism v7.0 was used to plot the SEC-MALS graph. OriginPro 2015 was used to plot the cell killing assay and mass spectrometry graphs. MicroCal PEAQ-ITC Analysis Software version 1.1.0.1262 was used to plot ITC graphs.

### Reporting summary

Further information on experimental design is available in the Nature Research Reporting Summary linked to this article.

## Supplementary information

Supplementary Information

Reporting Summary

Source Data

## Data Availability

Amino acid sequences of SpyDock and SpyCatcher002-oligomers are available in the Supplementary Information. Sequences of other constructs are available in GenBank as described above under “Plasmids and cloning”. Plasmids encoding the SpyDock and SpyCatcher002-oligomers were deposited in the Addgene repository (https://www.addgene.org/Mark_Howarth/). The source data for Fig. 2a, b, d, 3, 4, 5, 6b, c, 7b–d, and Supplementary Figs. 1, 3, 4, 5, 6, and 8b, c are provided as a Source Data File. Further information and request for resources and reagents should be directed to and will be fulfilled by the Lead Contact, Mark Howarth (mark.howarth@bioch.ox.ac.uk).
