# Supplementary material for: Spy&Go purification of SpyTag-proteins using pseudo-SpyCatcher to access an oligomerization toolbox
Source: Nat Commun. 2019 Apr 15;10:1734. doi: 10.1038/s41467-019-09678-w (PMC6465384; doi:10.1038/s41467-019-09678-w)
Supplement: Supplementary file 1 — Supplementary Information [file 41467_2019_9678_MOESM1_ESM.pdf]

Supplementary Information

Spy&Go purification of SpyTag-proteins using pseudo-SpyCatcher to access an oligomerization toolbox

Khairil Anuar et al.

Department of Biochemistry, University of Oxford, South Parks Road, Oxford OX1 3QU, UK

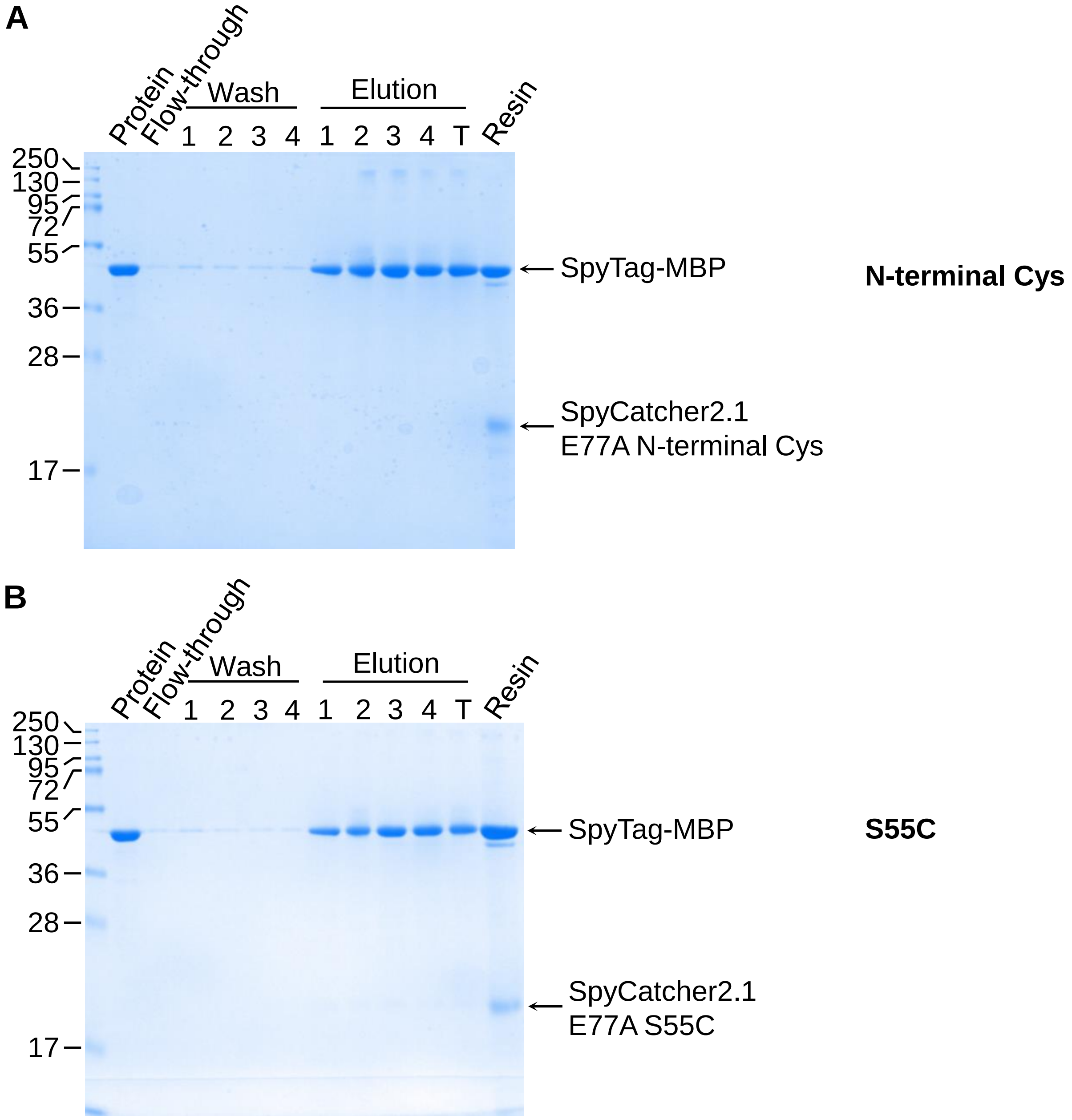

Supplementary Figure 1. Comparison of cysteine-anchoring residues for SpyDock. Purified SpyTag-MBP was mixed with SpyDock anchored to resin by different cysteine-anchoring residues **(A**: N-terminal Cys, **B**: S55C**)**. The sample was washed and eluted, before analysis by SDS-PAGE with Coomassie staining. Protein: input SpyTag-MBP protein. T: total pooled elutions.

```
                 1         10        20        30        40        50
SpyCatcher       AMVDTLSGLSSEQGQSGDMTIEEDSATHIKFSKRDEDGKELAGATMELRDSSGKTIS
SpyCatcher002    AMVTTLSGLSGEQGPSGDMTTEEDSATHIKFSKRDEDGRELAGATMELRDSSGKTIS
SpyDock          AMVTTLSGLSGEQGPSGDMTTEEDSATHIKFSKRDEDGRELAGATMELRDCSGKTIS

                     60        70        80        90        100       110
SpyCatcher       TWISDGQVKDFYLYPGKYTFVETAAPDGYEVATAITFTVNEQGQVTVNGKATKGDAHI*
SpyCatcher002    TWISDGHVKDFYLYPGKYTFVETAAPDGYEVATAITFTVNEQGQVTVNGEATKGDAHT*
SpyDock          TWISDGHVKDFYLYPGKYTFVATAAPDGYEVATPITFTVNEDGQVTVNGEATEGDAHT*

SpyTag           -AHIVMVDAYKPTK
SpyTag002        VPTIVMVDAYKRYK
```

Supplementary Figure 2. Amino acid sequence alignment of SpyCatcher, SpyCatcher002 and SpyDock, numbered according to the PDB structure 2X5P. SpyTag and SpyTag002 are also aligned. Underlined green shows mutations to make SpyDock, red shows mutations to make SpyCatcher002, and cyan shows mutations to make SpyTag002.

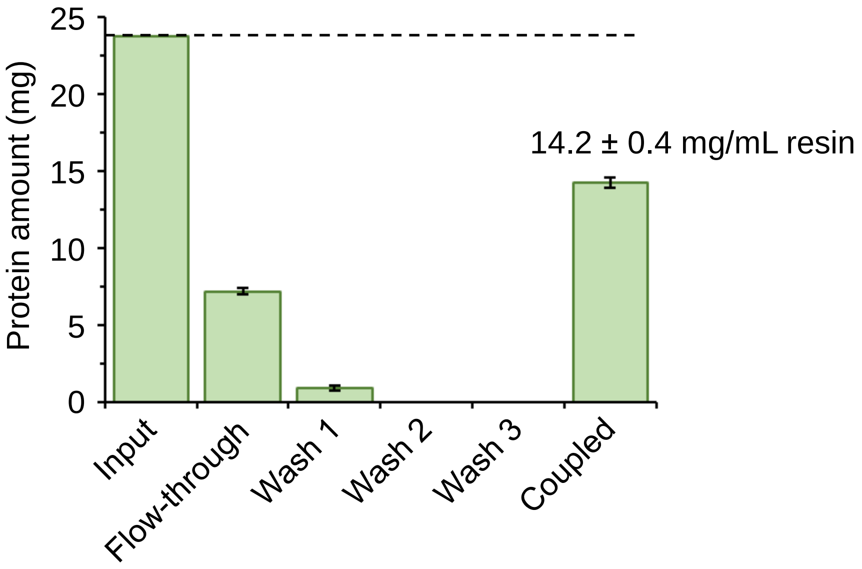

Supplementary Figure 3. Coupling efficiency of SpyDock to resin. SpyDock concentration and amount were determined by NanoDrop. 23.7 mg reduced SpyDock (Input) was coupled to 1.1 mL resin, with unbound protein removed as flow-through, wash 1, wash 2 and wash 3. The amount of SpyDock coupled (mean ± 1 s.d., $n = 3$) is equal to the protein amount in Input – (flow-through + wash 1 + wash 2 + wash 3). The dotted line is the threshold of input protein.

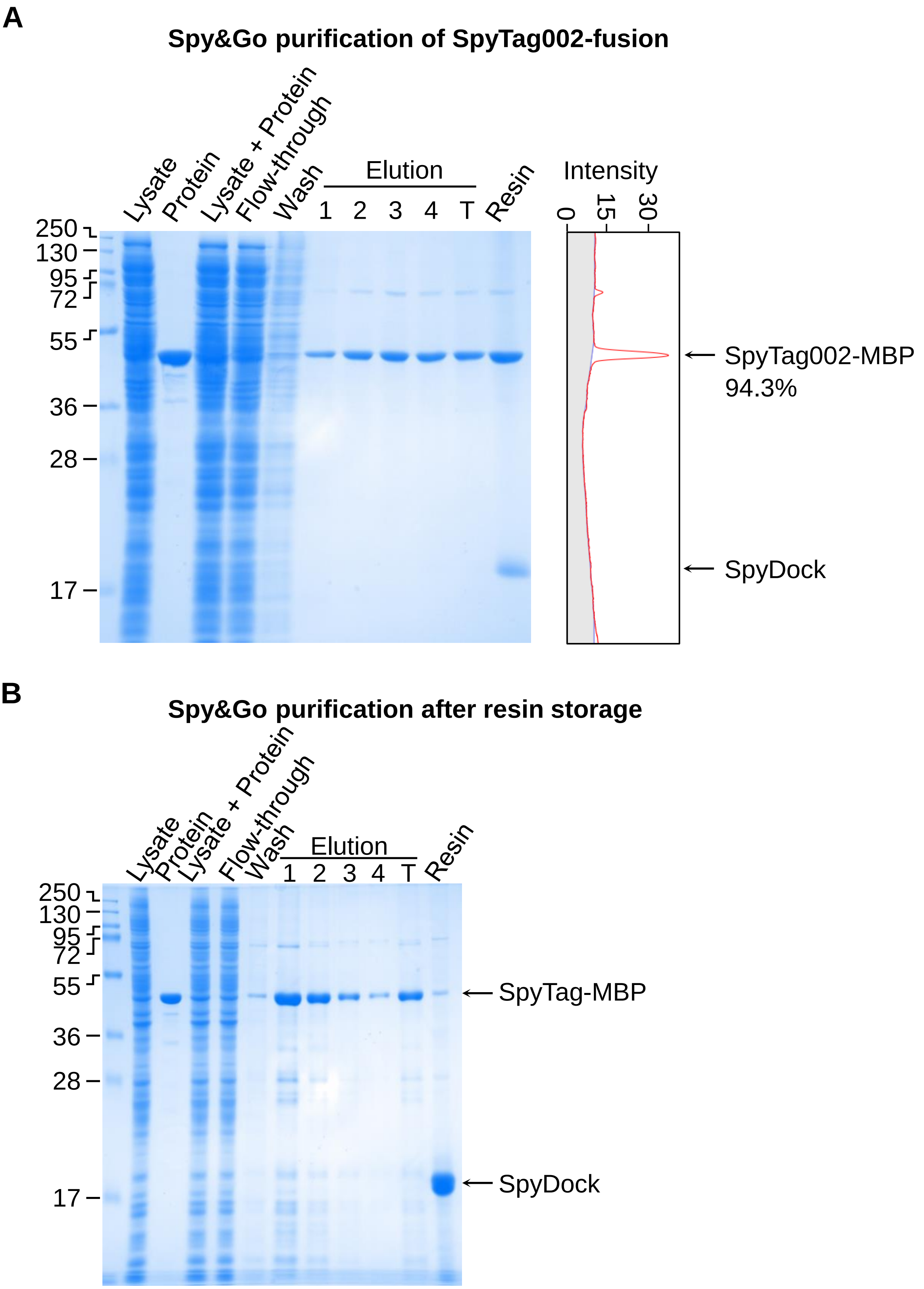

Supplementary Figure 4. Spy&Go purification is also possible with SpyTag002 and from 20% ethanol storage. **(A)** SpyTag002-MBP was purified from *E. coli* lysate by Spy&Go and analyzed by SDS-PAGE with Coomassie staining. **(B)** SpyTag-MBP was purified from *E. coli* lysate after Spy&Go resin was stored in 20% (v/v) ethanol for 12 weeks. Protein: input SpyTag-MBP protein. T: total pooled elutions. SDS-PAGE gel band scan of lane T shows the relative purity of Spy&Go-purified SpyTag002-MBP.

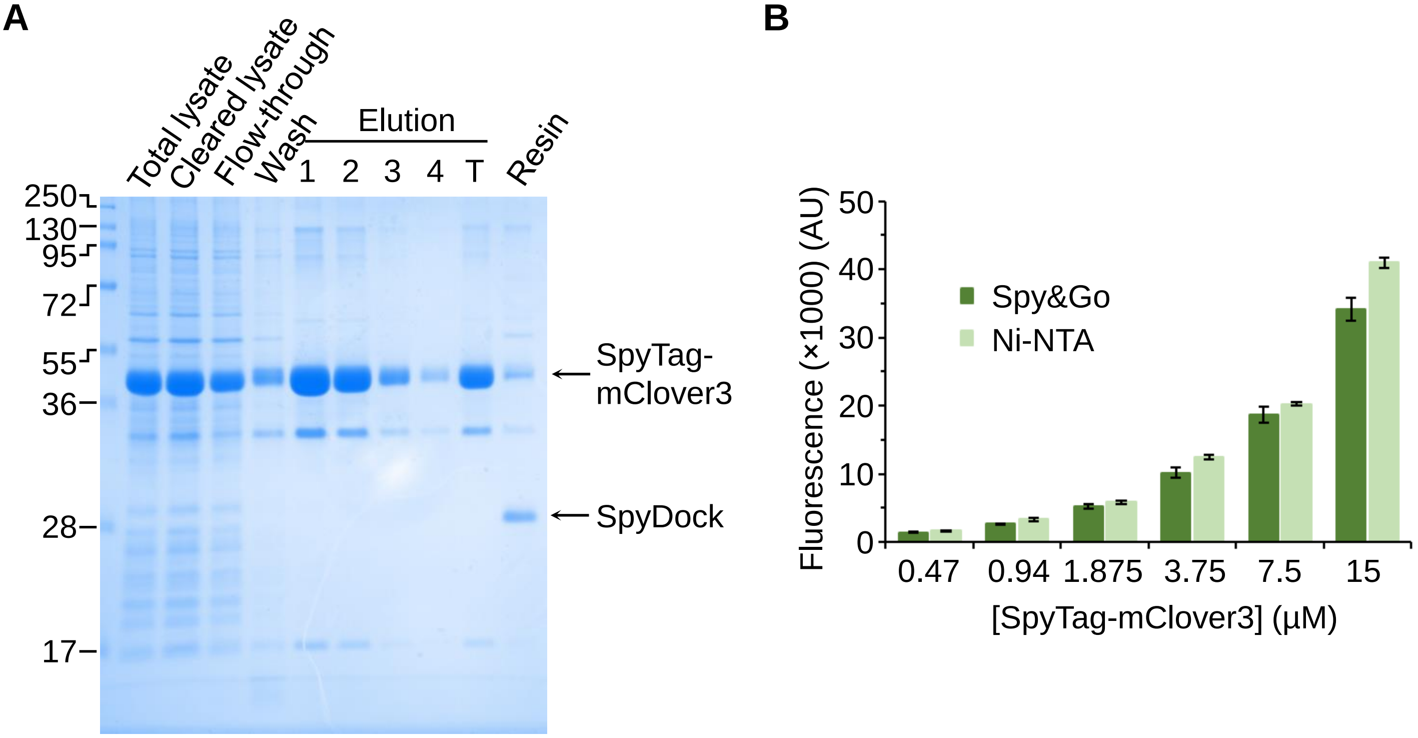

Supplementary Figure 5. Spy&Go purification of a fluorescent protein. **(A)** SpyTag-mClover3 was purified from *E. coli* lysate by Spy&Go and analyzed by SDS-PAGE with Coomassie staining. T: total pooled elutions. **(B)** Comparison of fluorescence of SpyTag-mClover3 purified by Spy&Go or Ni-NTA at different concentrations (mean ± 1 s.d., $n = 3$) (AU = arbitrary units).

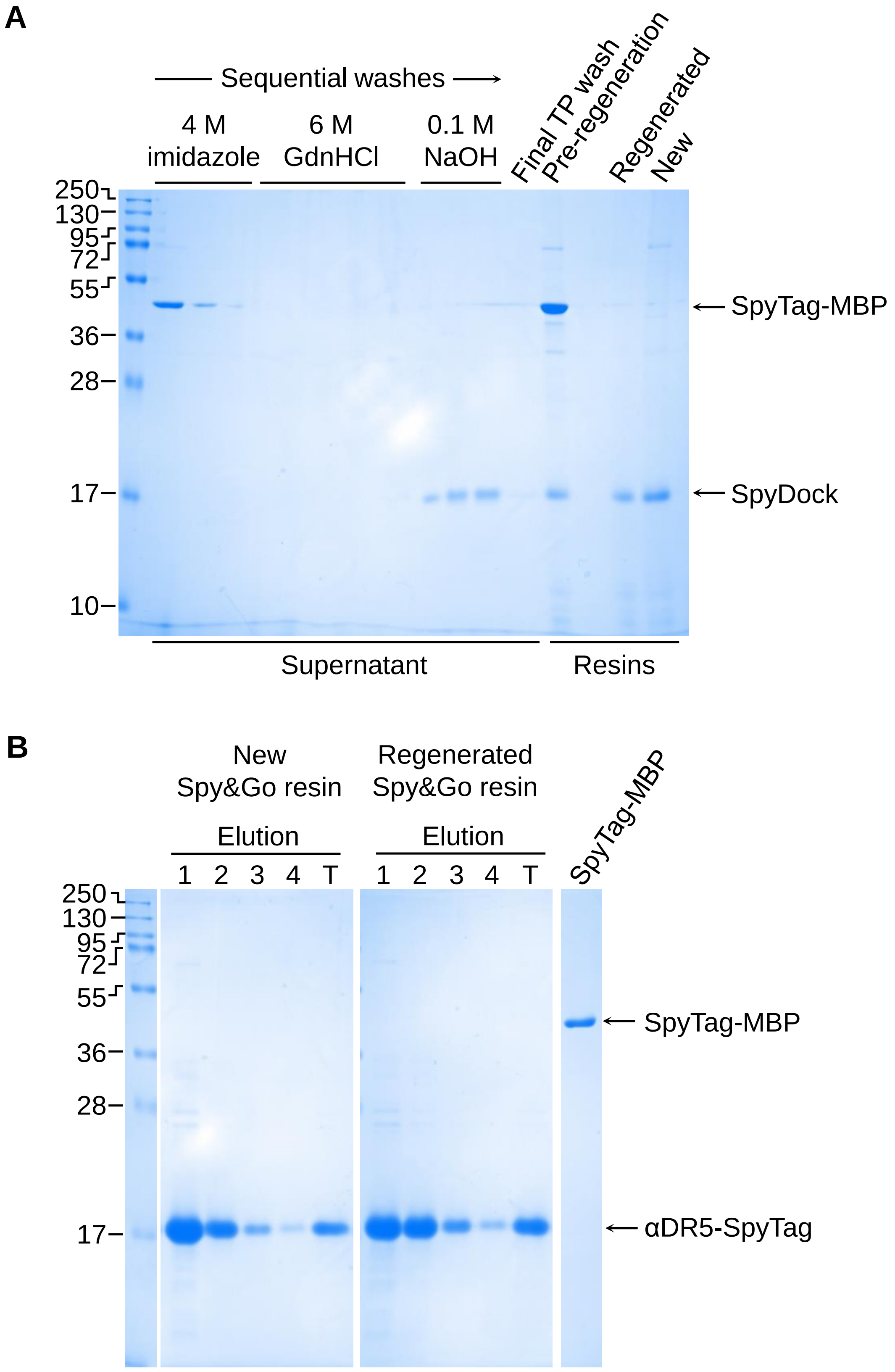

Supplementary Figure 6. Regeneration of Spy&Go resin. **(A)** Regeneration steps to remove residual SpyTag-MBP from a previous purification. "Pre-regeneration" shows the resin after SpyTag-MBP purification. "Regenerated" shows the resin following sequential imidazole, guanidinium hydrochloride and NaOH washes, in comparison to a "New" unused resin batch. **(B)** Spy&Go purification of αDR5-SpyTag using New unused resin or Regenerated resin from (A). Elution fractions 1 – 4 and the total pooled elution (T) fraction are shown. Data represent SDS-PAGE with Coomassie staining.

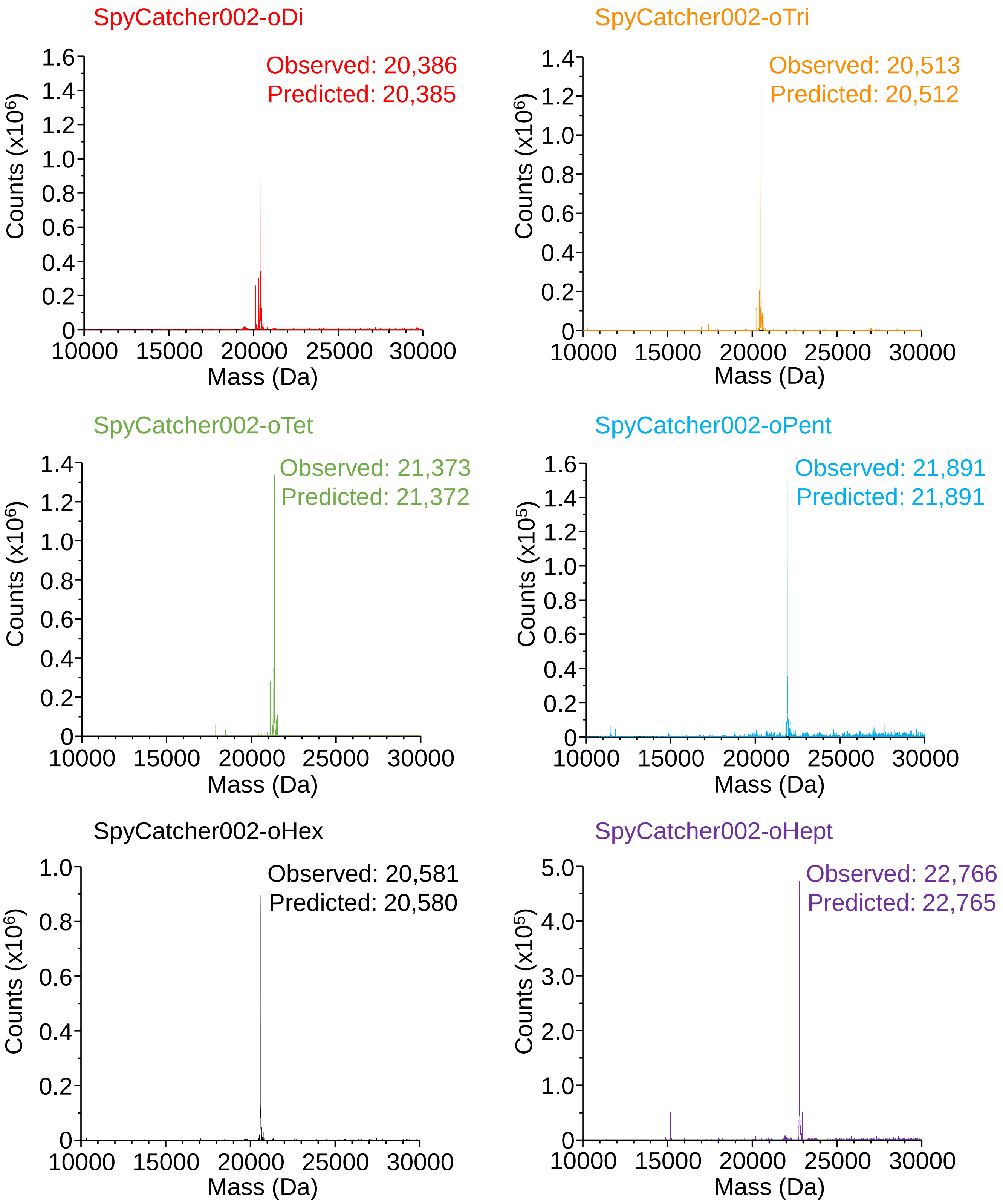

Supplementary Figure 7. Electrospray ionization mass spectra of SpyCatcher002-coiled coil subunits. The highest peak observed was compared to the predicted mass (Da).

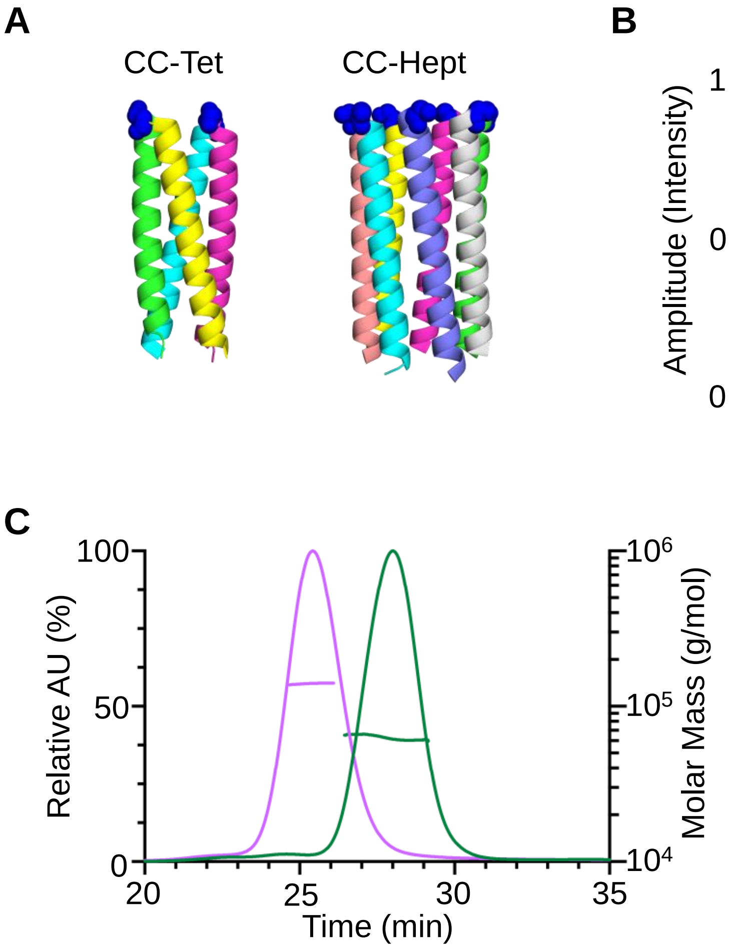

	Observed M_r	Expected M_r (kDa)
CC-Tet	62.8 ± 0.5	81.5
CC-Hept	140.0 ± 0.1	143.5

Supplementary Figure 8. Characterization of alternative coiled-coils for the Spy oligomerization toolbox. **(A)** CC-Tet and CC-Hept were evaluated for oTet and oHept. The coiled coils are shown with each color representing one chain and the N-terminus as a blue ball. **(B)** DLS of SpyCatcher002-CC-Tet and SpyCatcher002-CC-Hept, with hydrodynamic radius (R_h) (mean ± 1 s.d., *n* = 10) for each assembly. **(C)** SEC-MALS of SpyCatcher002-CC-Tet and SpyCatcher002-CC-Hept. The peak shows the normalized absorbance units (AU) at 280 nm of the SpyCatcher002-oligomer from SEC. The horizontal line shows the distribution of molar mass (g/mol) in the peak from MALS. Observed and expected molecular weights (M_r) are shown alongside, with error bars representing the uncertainty in fit to the molar mass curve.

SpyCatcher002-oDi

MSYYHHHHHHDYDIPTTENLYFQGAMVTTLSGLSGEQGPSGDMTTEEDSATHIKFSKRDEDGRELAGATM
ELRDSSGKTISTWISDGHVKDFYLYPGKYTFVETAAPDGYEVATAITFTVNEQGQVTVNGEATKGDAHTG
SSGSGSGSEIAALKKEIAALKQENAALKQEIAALKKEIAALKQGSGSGEPEA*

SpyCatcher002-oTri

MSYYHHHHHHDYDIPTTENLYFQGAMVTTLSGLSGEQGPSGDMTTEEDSATHIKFSKRDEDGRELAGATM
ELRDSSGKTISTWISDGHVKDFYLYPGKYTFVETAAPDGYEVATAITFTVNEQGQVTVNGEATKGDAHTG
SSGSGSGSEIAAIKKEIAAIKQEIAAIKQEIAAIKKEIAAIKQQGSGSGEPEA*

SpyCatcher002-oTet

MSYYHHHHHHDYDIPTTENLYFQGAMVTTLSGLSGEQGPSGDMTTEEDSATHIKFSKRDEDGRELAGATM
ELRDSSGKTISTWISDGHVKDFYLYPGKYTFVETAAPDGYEVATAITFTVNEQGQVTVNGEATKGDAHTG
SSGSGSGSSDYSDLQRVKQELLEEVKKELQKVKEEIIEAFVQELRKRGSGSGEPEA*

SpyCatcher002-oPent

MSYYHHHHHHDYDIPTTENLYFQGAMVTTLSGLSGEQGPSGDMTTEEDSATHIKFSKRDEDGRELAGATM
ELRDSSGKTISTWISDGHVKDFYLYPGKYTFVETAAPDGYEVATAITFTVNEQGQVTVNGEATKGDAHTG
SSGSGSGSMDLAPQMLRELQETNAALQDVRELLRQQVKEITFLKNTVMECDACGGSGSGEPEA*

SpyCatcher002-oHex

MSYYHHHHHHDYDIPTTENLYFQGAMVTTLSGLSGEQGPSGDMTTEEDSATHIKFSKRDEDGRELAGATM
ELRDSSGKTISTWISDGHVKDFYLYPGKYTFVETAAPDGYEVATAITFTVNEQGQVTVNGEATKGDAHTG
SSGSGSGSEIAKSLKEIAKSLKEIAWSLKEIAKSLKEIAWSLKGSGSGEPEA*

SpyCatcher002-oHept

MSYYHHHHHHDYDIPTTENLYFQGAMVTTLSGLSGEQGPSGDMTTEEDSATHIKFSKRDEDGRELAGATM
ELRDSSGKTISTWISDGHVKDFYLYPGKYTFVETAAPDGYEVATAITFTVNEQGQVTVNGEATKGDAHTG
SSGSGSGSKKQGDADVCGEVAYIQSVVSDCHVPTAELRTLLEIRKLFLEIQKLKVELQGLSKEGSGSGEP
EA*

Supplementary Figure 9: Amino acid sequences of the Spy oligomerization toolbox. The His-tag is colored in green, TEV cleavage site is underlined, SpyCatcher002 is colored in blue, coiled-coil in red, and C-tag in yellow. * represents the stop codon.
